# Five 2-(2-Phenylethyl)chromones from Sodium Chloride-Elicited *Aquilaria sinensis* Cell Suspension Cultures

**DOI:** 10.3390/molecules21050555

**Published:** 2016-04-27

**Authors:** Zhongxiu Zhang, Xiaohui Wang, Wanqing Yang, Juan Wang, Cong Su, Xiao Liu, Jun Li, Yunfang Zhao, Shepo Shi, Pengfei Tu

**Affiliations:** 1 Modern Research Center for Traditional Chinese Medicine, Beijing University of Chinese Medicine, Beijing 100029, China; zhangzx07@163.com (Z.Z.); wangxhui2014@163.com (X.W.); yangwanqing511@163.com (W.Y.); wjwangjuan2012@163.com (J.W.); suerzong@163.com (C.S.); fcliuxiao@163.com (X.L.); drlj666@163.com (J.L.); yunfang.zhao@163.com (Y.Z.); 2 School of Chinese Materia Medica, Beijing University of Chinese Medicine, Beijing 100102, China

**Keywords:** 2-(2-phenylethyl)chromone, induced circular dichroism, *Aquilaria sinensis*, agarwood, cell suspension cultures, sodium chloride elicitation

## Abstract

Five 2-(2-phenylethyl)chromones including a new one, (5*S*,6*R*,7*S*,8*R*)-5,8-dichloro-6,7-dihydroxy-2-phenylethyl-5,6,7,8-tetrahydro-4*H*-chromen-4-one (**1**), and four known ones (**2**–**5**), were isolated from 150 mM NaCl-elicited *Aquilaria sinensis* cell suspension cultures. In addition, three feruloyl amides (**6**–**8**), six nucleosides (**9**–**14**), (+)-syringaresinol (**15**), indole-3-carboxaldehyde (**16**), and two glycosides (**17**–**18**) were also obtained. The structures were unambiguously identified by analysis of their UV, IR, NMR, and HRESIMS data. The absolute configuration of the new 2-(2-phenylethyl)chromone (**1**) was established by a dimolybdenum tetraacetate-induced circular dichroism experiment. Compared to un-elicited cell lines, the appearance of 2-(2-phenylethyl)chromones in NaCl-treated cells occurred on the 3rd and 5th days of their treatment. 2-(2-Phenylethyl)chromones, feruloyl amides, nucleosides, and lignins have been reported to be closely related to plant defense; therefore, the identification of these compounds from NaCl-elicited *A. sinensis* cell suspension cultures would be useful for further exploring the mechanism of agarwood formation.

## 1. Introduction

2-(2-Phenylethyl)chromones are a subgroup of plant polyphenols specifically produced by *Aquilaria*, *Gonystulus*, and *Gyrinops* species (Thymelaeaceae) in response to biotic or abiotic stress. Hitherto, more than 100 congeners of 2-(2-phenylethyl)chromones have been reported, and many of them have potentially anti-inflammatory [1], neuroprotective [2], antitumor activities, *etc.* [3,4,5]. Under stress conditions, such as wounding or fungal infections, some resin-impregnated heartwood containing a variety of 2-(2-phenylethyl)chromones are slowly forming in the trunk and branches of some species of Thymelaeaceae. Those resinous heartwoods are commercially called agarwood, which have long been used pharmaceutically as a digestive, sedative, and antiemetic in oriental medical treatments [6] and as incense in many cultures because of its unique fragrance. The finest agarwood pieces are considerably valued in some Asian incense ceremonies. An extremely long growing cycle and an increased level of consumption are probably the main reasons of the over-exploitation and serious depletion of agarwood, leading to the relative rarity and high cost of its wild resource. Since 2005, accordingly, *Aquilaria* and *Gyrinops* have been listed in Appendix II (potentially threatened species) of the Convention on International Trade in Endangered Species of Wild Fauna and Flora [7]. Hence, it should be of great interest to unveil the mechanisms of agarwood formation and thereby to artificially manipulate the process of agarwood formation [8].

Because it takes timber species a considerably long time for their resinous portions to form inside of the wood of agarwood-producing plants, researchers tend to use cell suspension cultures to study the mechanisms of agarwood formation. Therefore, establishing cell suspension cultures with a high rate of cell growth and a high production of 2-(2-phenylethyl)chromones, the characteristic components of agarwood, would be undoubtedly meaningful. Here, cell suspension cultures of *Aquilaria sinensis*, the only origin species of agarwood in China, were established, and the chemical constituents of NaCl-elicited *A. sinensis* cell suspension cultures were investigated, resulting in the isolation of five 2-(2-phenylethyl)chromones (**1**–**5**) together with 13 other known compounds (**6**–**18**) (Figure S1). The structural elucidation of the new 2-(2-phenylethyl)chromone (**1**) is comprehensively discussed herein.

## 2. Results and Discussion

**Figure 1:**
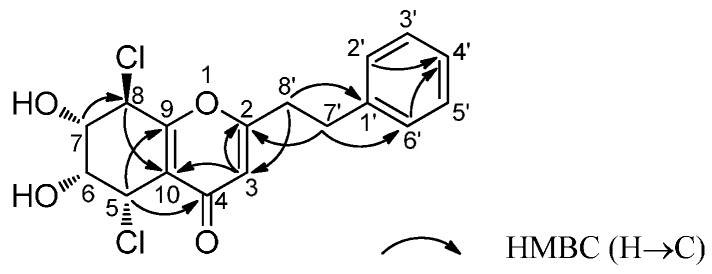
Selected HMBC correlations of Compound **1**.

Compound **1** was obtained as a brown amorphous powder. ${\left[\alpha \right]}_{D}^{25}$ +19.0° (*c* = 0.03, MeOH). The HRESIMS spectrum showed the presence of a protonated molecule peak [M + H]^+^ at *m*/*z* 355.0484, in accordance with a empirical molecular formula C_17_H_16_O_4_Cl_2_ (calcd for C_17_H_16_O_4_Cl_2_, 355.0498). The ratio of [M + H]^+^ isotope peaks, 3:2, at *m*/*z* 355/357 clearly indicated the presence of two chlorine atoms in **1**. In the IR spectrum of **1**, absorptions at 3417 cm^−1^ and 1659 cm^−1^ suggested the presence of hydroxyl groups and carbonyl group in **1**. The ^1^H-NMR spectrum of **1** showed the presence of a phenylethyl moiety at *δ*_H_ 7.19–7.29 (5H, m), 2.94–3.04 (4H, m), a singlet at *δ*_H_ 6.17(1H, s), and four consecutive methine protons at *δ*_H_ 4.94 (1H, br.s), 4.19 (1H, br.s), 4.36 (1H, br.d, *J* = 8.0 Hz), and 4.95 (1H, d, *J* = 8.0 Hz). The ^13^C-NMR exhibited the presence of 17 carbons including two methylenes at *δ*_C_ 33.8 and 36.2, and four methines at *δ*_C_ 52.0, 58.3, 72.8, and 74.3. Comparison of the NMR data with those of the known Compound **2** [9] revealed that these two compounds shared a very similar skeleton. The only difference was the presence of two methines at a relatively higher field (*δ*_C_ 52.0, 58.3) in the ^13^C-NMR spectrum of **1**, suggesting these two carbons were both chlorinated. In the HMBC spectrum, the long range correlations between the proton at *δ*_H_ 4.94 (1H, br.s), which was attached at C-5 (*δ*_C_ 52.0), and the carbonyl carbon at *δ*_C_ 179.4 definitively established that one of the two chlorine atoms were attached at C-5. The other chlorine atom at C-8 was deduced from the HMBC correlations of H-8/C-10 and H-7/C-8 (Figure 1). Accordingly, the planar structure of **1** was elucidated as 5,8-dichloro-6,7-dihydroxy-2-phenylethyl-5,6,7,8-tetrahydro-4*H*-chromen-4-one (see Figures S2–S9).

The relative configuration of **1** was established by analysis of the coupling constants between the involved protons and further confirmed by a NOESY experiment. In the ^1^H-NMR spectrum, the relatively large coupling constant between H-7 and H-8 (*J* = 8.0 Hz) revealed the *trans* pseudoaxial-axial relationships between H-7 and H-8. In contrast, the relatively small coupling constants between H-5, H-6, and H-7 revealed the *cis* relationships between these protons, which were confirmed by the NOE correlations of H-5/H-6, H-5/H-7 in the NOESY spectrum of **1**.

**Figure 2:**
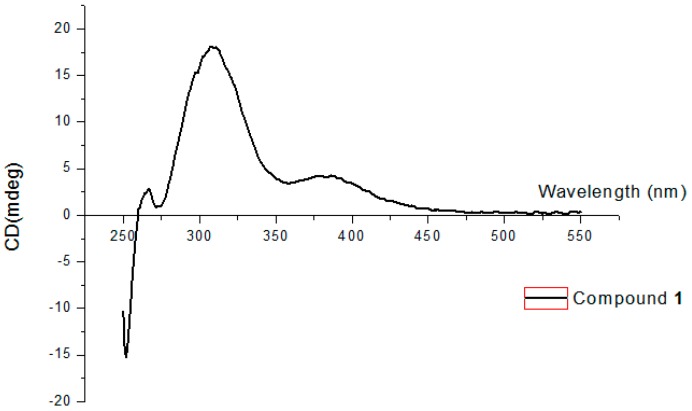
Induced circular dichroism (ICD) spectrum of Compound **1** in solution of Mo_2_(AcO)_4_.

The absolute configurations of C-6 and C-7 were solved according to the method reported by Snatzke and Frelek [10]. DMSO solutions of **1** and dimolybdenum tetraacetate [Mo_2_(AcO)_4_] were homogeneously mixed, and the induced circular dichroism (ICD) spectrum was recorded. In the ICD spectrum of **1** (Figure 2), the positive Cotton effect at 310 nm suggested a positive dihedral angle of the O−C−C−O moiety, which allowed to the assignment of the absolute configuration of C-6 and C-7 as *R* and *S*, respectively. Thus, the absolute configuration of C-5 and C-8 was assigned as *S* and *R*. Accordingly, Compound **1** was elucidated as (5*S*,6*R*,7*S*,8*R*)-5,8-dichloro-6,7-dihydroxy-2-phenylethyl-5,6,7,8-tetrahydro-4*H*-chromen-4-one (Figure 1).

By comparison of their MS and NMR data with those reported, the known 2-(2-phenylethyl)chromones (**2**–**5**) and other 13 known compounds (**6**–**18**) were identified as: (5*S*,6*S*,7*S*,8*R*)-8-chloro-5,6,7-trihydroxy-2-phenylethyl-5,6,7,8-tetrahydro-4*H*-chromen-4-one (**2**) [9], (5*S*,6*R*,7*R*,8*S*)-8-chloro-5,6,7-trihydroxy-2-phenethyl-5,6,7,8-tetrahydro-4*H*-chromen-4-one (**3**) [11], 6,7-dimethoxy-2-(2-phenylethyl) chromone (**4**) [12], 6,7-dimethoxy-2-[2-(4-methoxyphenyl) ethyl] chromone (**5**) [13], *N-trans*-feruloyltyramine (**6**) [14], *N-trans*-feruloyloctopamine (**7**) [14], *N-cis*-feruloyltyramine (**8**) [14], *N*^6^-methyladenosine (**9**) [15], adenosine (**10**) [16], 2’-deoxy-d-adenosine (**11**) [16], thymidine (**12**) [17], 2’-deoxyuridine (**13**) [17], uridine (**14**) [18], (+)-syringaresinol (**15**) [19], indole-3-carboxaldehyde (**16**) [20], 4-hydroxyl-3,5-dimethoxyl-6-*O-*β*-*d-glucosebenzene (**17**) [21], and 6-*O*-acetyl β*-*d-glucopyranose (**18**) [22] (Figure S1).

It is well known that biotic and abiotic stresses could adversely affect plant growth and subsequently cause the production of specific secondary metabolites for plant defense. As the characteristic constituents and parts of the principal fragrant compounds of agarwood, 2-(2-phenylethyl)chromones tend to be formed by stressed *A. sinensis*, but not by healthy intact *A. sinensis*, suggesting that 2-(2-phenylethyl)chromones are closely related to plant defense. On the other hand, 2-(2-phenylethyl)chromones could be detected from salicylic acid, or fungal extract-induced cell suspension cultures of *A. sinensis* [23,24]. In this report, NaCl was firstly used as an elicitor to induce cell suspension cultures of *A. sinensis* to produce 2-(2-phenylethyl)chromones, and the chlorinated 2-(2-phenylethyl)chromones could not be produced by using previously reported methods. Moreover, 2-(2-phenylethyl)chromones produced by fungal extract-induced cells are all flindersia-type (FDC-type) chromones, like Compounds **4** and **5** [24]. In contrast, NaCl-elicited cells produce both chlorinated tetrahedrochromones (**1**–**3**) and FDC-type chromones (**4**, **5**). According to our time course analysis on before- and after-elicitation experiments, **5** was first detected in the 3-day NaCl-elicited sample, and **1–4** were first detected in the 5-day NaCl-elicited sample (see Figures S10 and S11). The appearance of FDC-type chromone **5** occurred two days earlier than that of **1**–**4**. Additionally, feruloyl amides, nucleosides, and lignins have been extensively reported as being involved in plant defenses [25,26,27,28]. The identification of 2-(2-phenylethyl)chromones (**1**–**5**), feruloyl amides (**6**–**8**), nucleosides (**9**–**14**), and (+)-syringaresinol (**15**) would be useful for further exploring the mechanism of agarwood formation.

## 3. Materials and Methods

### 3.1. General Experimental Procedures

UV spectra were obtained using a Shimadzu UV-2450 spectrophotometer (Shimadzu Corporation, Tokyo, Japan). NMR spectra were recorded on a Varian INOVA-500 spectrometer (Varian Corporation, Palo Alto, CA, USA) operating at 500 MHz for ^1^H-NMR and 125 MHz for ^13^C-NMR. HRESIMS was recorded on an LCMS-IT-TOF system, fitted with a Prominence UFLC system and an ESI interface (Shimadzu Corporation). Optical rotations were obtained on a Rudolph Autopol IV automatic polarimeter (Rudolph Corporation, Flanders, NJ, USA). IR spectra were recorded on a Thermo Nicolet Nexus 470 FT-IR spectrophotometer (Thermo Corporation, Waltham, MA, USA) with KBr pellets. CD spectra were recorded using a Jasco J810 spectropolarimeter (YMC Corporation, Kyoto, Japan). HPLC was performed on a Shimadzu LC-20AT pump system (Shimadzu Corporation), equipped with a SPD-M20A photodiode array detector. A semi-preparative HPLC column (YMC-Pack C_18_, 250 × 10 mm, 5 μm, YMC Corporation) was employed for the isolation. TLC was performed using GF_254_ plates.

### 3.2. Cell Culture and Viability

Fresh young leaves of *A. sinensis* were cut into pieces, surface-sterilized using sodium hypochlorite (2.5% *v*/*v*) for 10 min, and rinsed with sterile distilled water four times. These processed leaves were then inoculated onto solid Murashige-Skoog (MS) basal media. Calli were initiated from the plant tissues after incubation in dark conditions for one month at 25 °C, subcultured in a MS liquid medium with 2 mg/L naphthal acetic acid, 1 mg/L 6-benzyladenin, 1 mg/L kinetin, 1 mg/L vitamin B5, and 1 mg/L 2,4-dichlorophenoxyacetic acid, and supplemented with sucrose (4% *w*/*v*) as a carbon source. The cells were cultivated via shaking at 250 rpm at 25 °C in the dark. The cell suspension cultures were subcultured into fresh medium every two weeks.

### 3.3. Elicitation and Harvesting Cells

Cell suspensions cultured for two weeks (5.28 L) were treated with NaCl (Beijing Chemicals, Beijing, China) at a final concentration of 150 mM. Control cell suspensions received no treatment. After different times of NaCl treatment, cells were harvested by filtration.

### 3.4. Extraction and Isolation

The filtrates were extracted with equivalent EtOAc at room temperature three times, whereas the cells (654.5 g) were extracted with MeOH (3.5 L) by sonication (30 min). The extracts were combined and concentrated under reduced pressure. The dried residue (17.6 g) was subjected to silica gel column chromatography and eluted with a stepwise gradient of CHCl_3_/MeOH (20:1 → 1:1, *v*/*v*) to afford four fractions (Fr. I–IV). Fr. I was separated by using semi-preparative HPLC (YMC-Pack C_18_, 250 × 10 mm, 5 μm, 65% aqueous MeOH) to yield **4** (1.2 mg, t_R_ 42 min), **5** (1.0 mg, t_R_ 36 min), and **16** (2.2 mg, t_R_ 10 min ). Fr. II was separated by using semi-preparative HPLC (YMC-Pack C_18_, 250 × 10 mm, 5 μm, 60% aqueous MeOH) to yield **1** (1.5 mg, t_R_ 33 min), **6** (1.9 mg, t_R_ 10 min), **7** (0.6 mg, t_R_ 9 min), **8** (1.8 mg, t_R_ 12 min), and **15** (1.1 mg, t_R_ 11 min). Fr. III was separated by using semi-preparative HPLC (YMC-Pack C_18_, 250 × 10 mm, 5 μm, 42% aqueous MeOH) to yield **2** (0.9 mg, t_R_ 22 min) and **3** (0.7 mg, t_R_ 10 min). Fr. IV was separated by using semi-preparative HPLC (YMC-Pack C_18_, 250 × 10 mm, 5 μm, 20% aqueous MeOH) to yield **9** (0.8 mg, t_R_ 20 min), **10** (5.7 mg, t_R_ 16 min), **11** (0.4 mg, t_R_ 18 min), **12** (4.8 mg, t_R_ 13 min), **13** (2.8 mg, t_R_ 9 min), **14** (4.2 mg, t_R_ 8min), **17** (3.0 mg, t_R_ 14 min), and **18** (2.2 mg, t_R_ 7 min).

*(5S,6R,7S,8R)-5,8-Dichloro-6,7-dihydroxy-2-phenylethyl-5,6,7,8-tetrahydro-4H-chromen-4-one* (**1**): Brown amorphous powder; ${\left[\alpha \right]}_{D}^{25}$ +19.0° (c = 0.03, MeOH); UV λ_max_(MeOH) 254 nm; IR (KBr): 3417, 2926, 2856, 1659, 1619, 1495, 1429, 1377, 1193, 1097 cm^−1^; HRESIMS *m*/*z*: 355.0484 [M + H]^+^ (calcd for C_17_H_16_O_4_Cl_2_: 355.0498); ^1^H-NMR (methanol-d_4_, 500 MHz) and ^13^C-NMR (methanol-*d*_4_, 125 MHz) data, see Table 1.

### 3.5. Absolute Configuration of Compound ***1***

According to the published procedure [10], Compound **1** (0.27 mg) was added to a stock solution of 0.6–0.7 mg/mL Mo_2_(AcO)_4_ (Strem Chemicals, Newburyport, MA, USA) in absolute anhydrous dimethylsulfoxide (DMSO) to achieve the ligand-to-metal ratio is approximately 0.6/0.8 up to 1.0/1.2. CD spectra of Compound **1** were recorded using a Jasco J810 spectropolarimeter, with a 0.1-cm cell in DMSO (Aladdin, Shanghai, China) and at room temperature, see Figure 2.

## 4. Conclusions

We have firstly reported five 2-(2-phenylethyl)chromones including a new one, together with 13 other known compounds from NaCl-elicited cell suspension cultures of *A. sinensis*. Most of these compounds have been previously demonstrated to be closely related to plant defense. Thus, it would be meaningful to further explore and understand the mechanism of pharmaceutically important and commercially valued agarwood formation.

## Figures and Tables

**Table 1:** ^1^H-NMR and ^13^C-NMR spectroscopic data of Compound **1** (*J* in Hz) ^a^.

No.	^1^H-NMR	^13^C-NMR
2		171.4
3	6.17 (1H, s)	114.2
4		179.4
5	4.94 (1H, br.s)	52.0
6	4.19 (1H,br.s)	74.3
7	4.36 (1H, br.d, *J* = 8.0 Hz)	72.8
8	4.95 (1H, d, *J* = 8.0 Hz)	58.3
9		161.7
10		121.8
1'		141.0
2', 6'	7.21 (2H, m, overlapped)	129.6
3', 5'	7.27 (2H, m, overlapped)	129.4
4'	7.19 (1H, m, overlapped)	127.6
7'	2.98 (2H, m, overlapped)	33.8
8'	3.03 (2H, m, overlapped)	36.2

^a^ ^1^H-NMR and ^13^C-NMR spectra were measured in methanol-*d*_4_ at 500 MHz and 125 MHz, respectively.

## Supplementary Materials

Supplementary materials can be accessed at: http://www.mdpi.com/1420-3049/21/5/555/s1.
