# Supplementary material for: Five 2-(2-Phenylethyl)chromones from Sodium Chloride-Elicited Aquilaria sinensis Cell Suspension Cultures
Source: Molecules. 2016 Apr 27;21(5):555. doi: 10.3390/molecules21050555 (PMC6274510; doi:10.3390/molecules21050555)
Supplement: Supplementary file 1 [file molecules-21-00555-s001.pdf]

Supplementary Materials: Five 2-(2-Phenylethyl)chromones from Sodium Chloride-Elicited *Aquilaria sinensis* Cell Suspension Cultures

Zhongxiu Zhang, Xiaohui Wang, Wanqing Yang, Juan Wang, Cong Su, Xiao Liu, Jun Li, Yunfang Zhao, Shepo Shi and Pengfei Tu

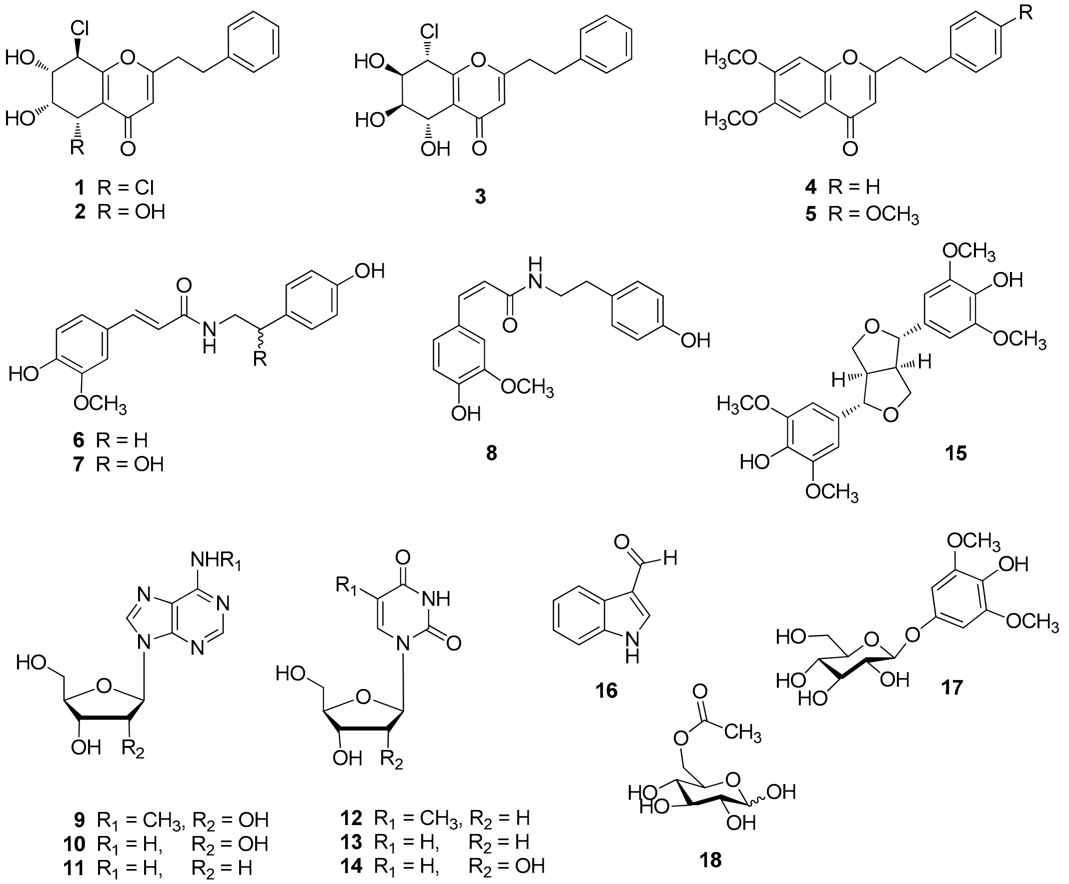

Figure S1. Structures of Compounds **1–18**.

Formula Predictor Report - Compound 1.lcd Page 1 of 1

Data File: D:\Datas\张钟秀\Compound 1.lcd

Elmt	Val.	Min	Max	Elmt	Val.	Min	Max	Elmt	Val.	Min	Max	Use Adduct
H	1	0	20	O	2	0	5	S	2	0	0	H
C	4	0	20	F	1	0	0	Cl	1	0	2	
N	3	0	0	P	3	0	0					

Error Margin (ppm):	5	DBE Range:	-2.0 - 1000.0	Electron Ions:	both
HC Ratio:	unlimited	Apply N Rule:	no	Use MSn Info:	yes
Max Isotopes:	all	Isotope RI (%):	1.00	Isotope Res:	10000
MSn Iso RI (%):	75.00	MSn Logic Mode:	AND	Max Results:	100

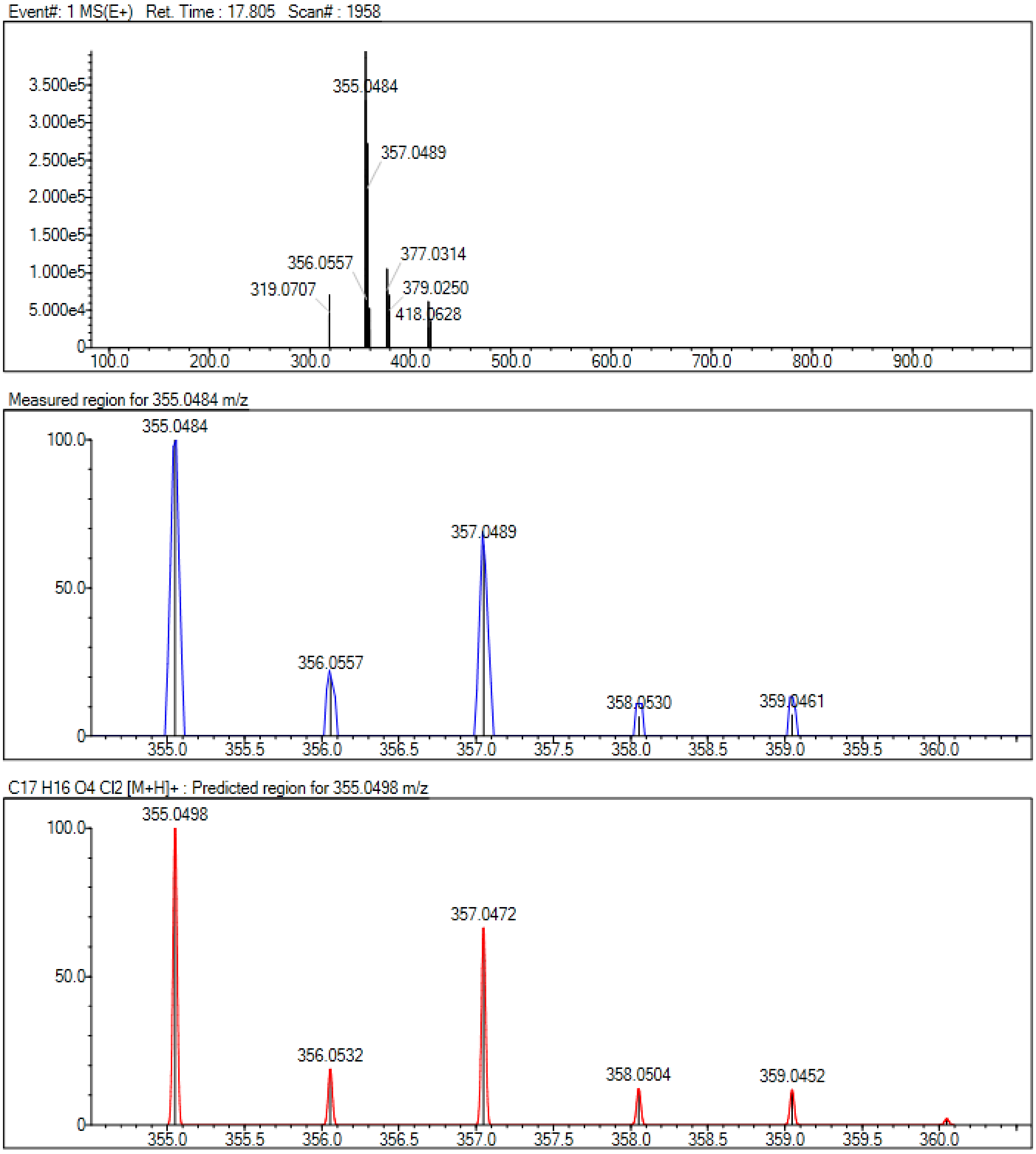

Rank	Score	Formula (M)	Ion	Meas. m/z	Pred. m/z	Df. (mDa)	Df. (ppm)	Iso	DBE
1	92.65	C17 H16 O4 Cl2	[M+H]+	355.0484	355.0498	-1.4	-3.94	100.00	9.0

Figure S2. HRESIMS spectrum of Compound **1**.

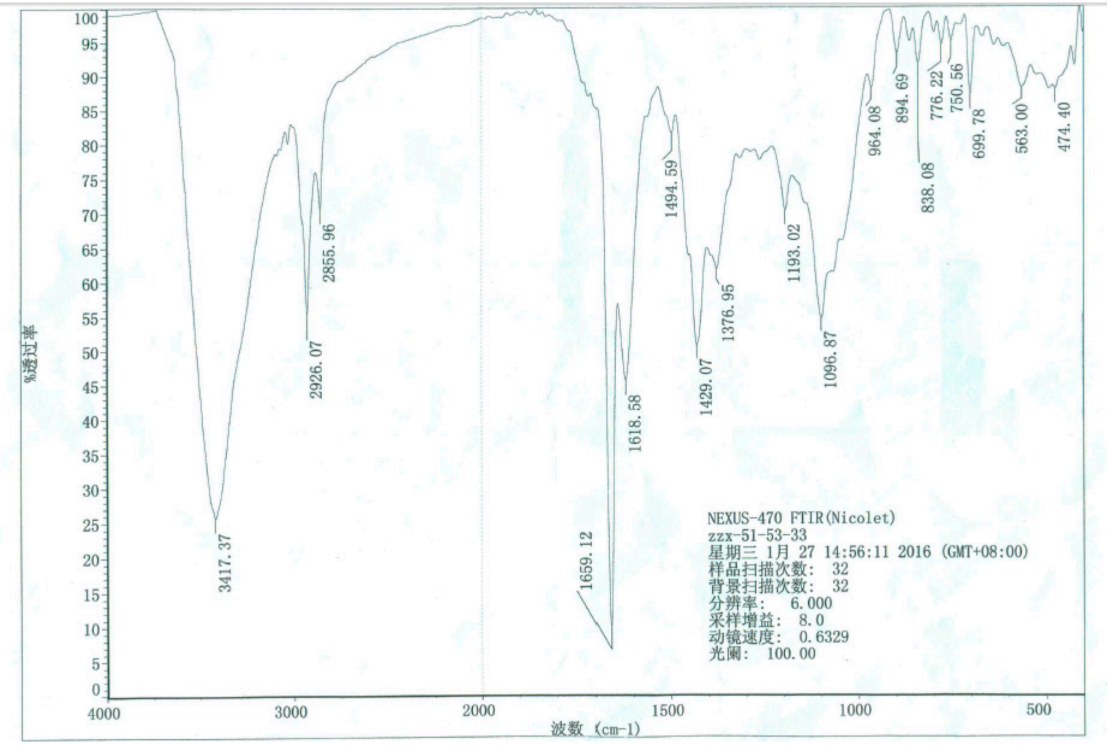

Figure S3. IR spectrum of Compound **1**.

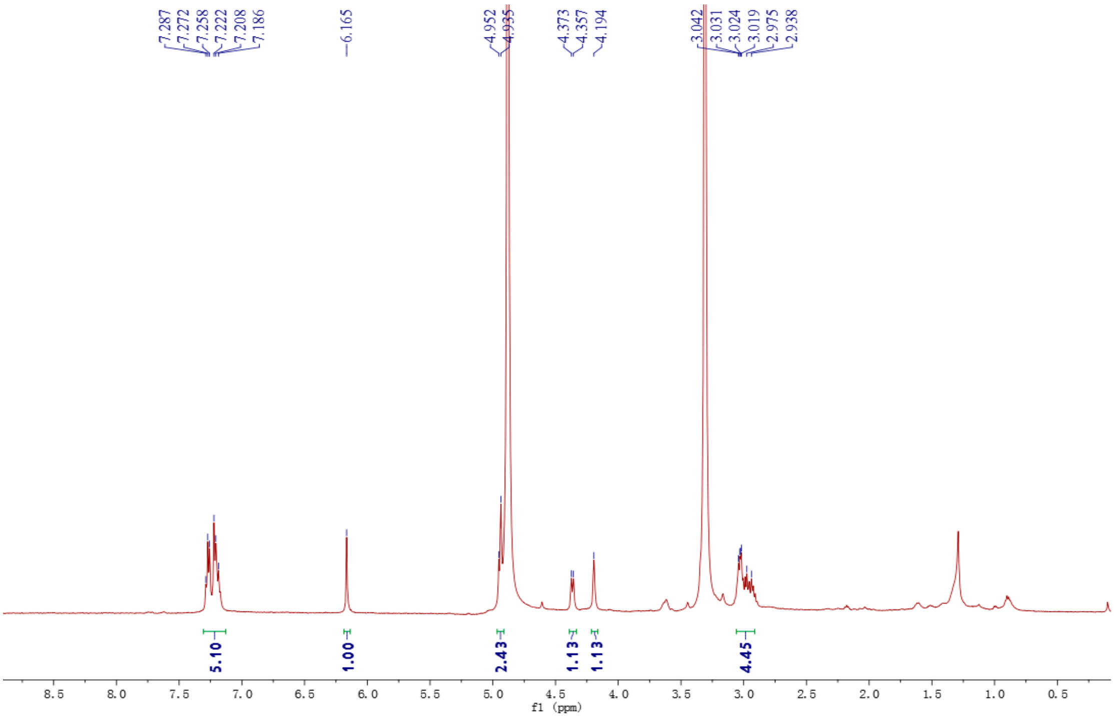

Figure S4. ^{1}H NMR spectrum of Compound **1** (CD_3OD, 500 MHz).

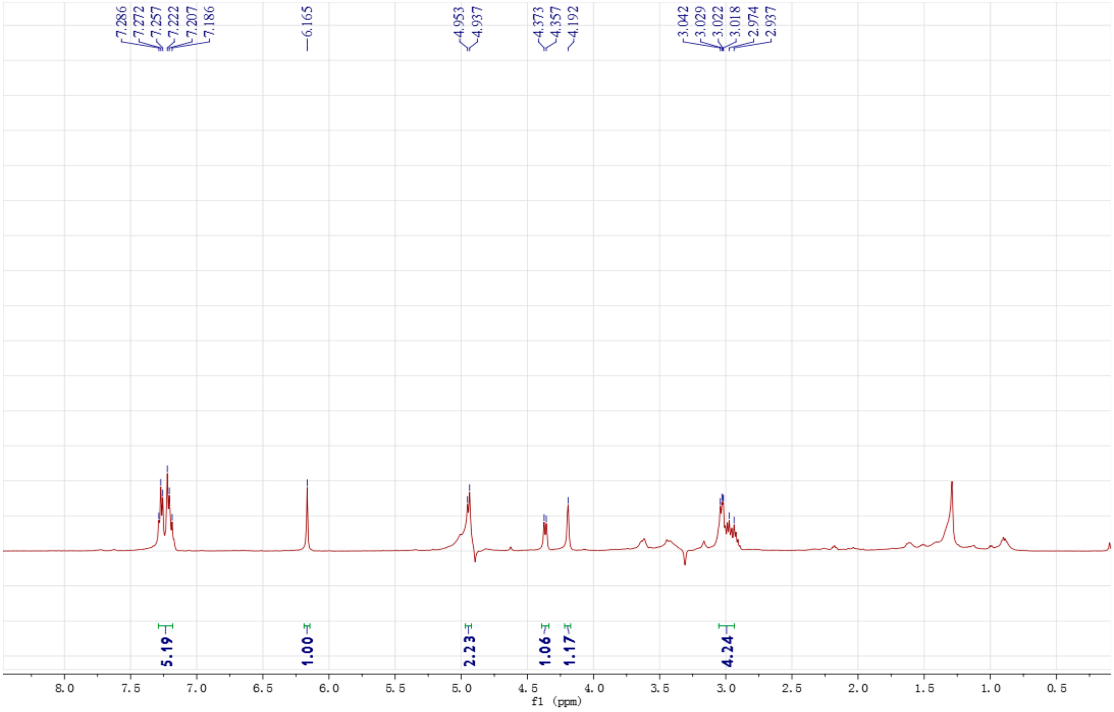

Figure S5. WET-¹H NMR spectrum of Compound **1** (CD_3OD, 500 MHz).

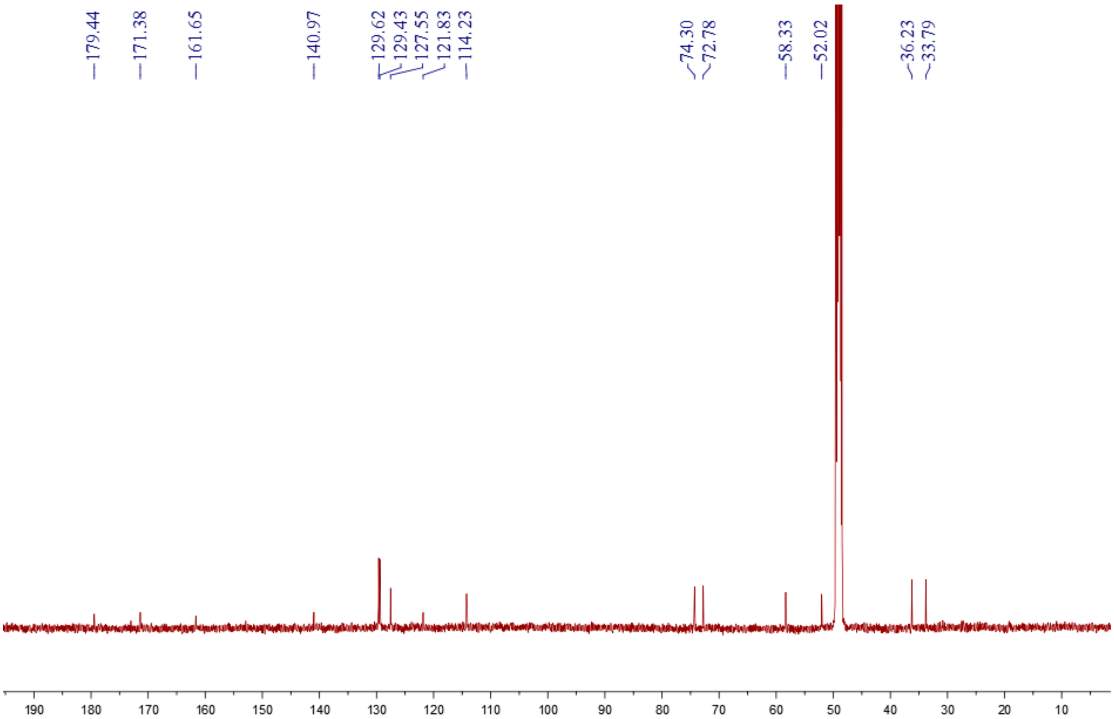

Figure S6. ^{13}C-NMR spectrum of Compound **1** (CD_3OD, 125 MHz).

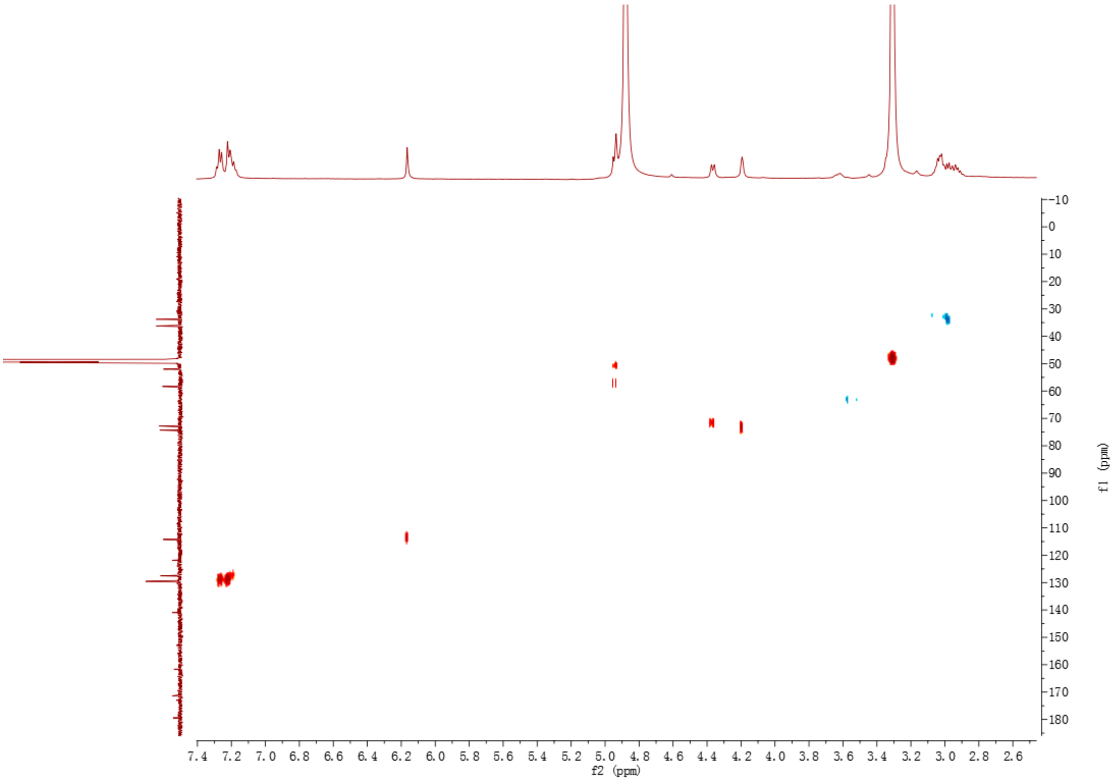

Figure S7. gHSQC spectrum of Compound **1**.

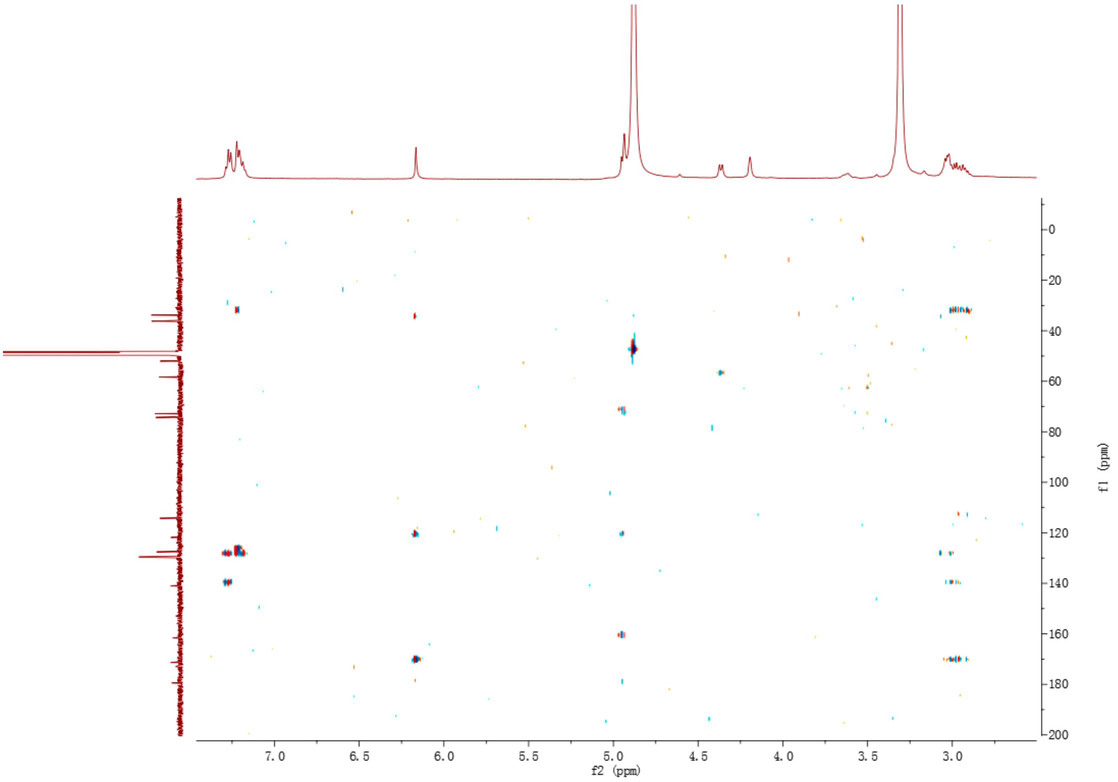

Figure S8. gHMBC spectrum of Compound **1**.

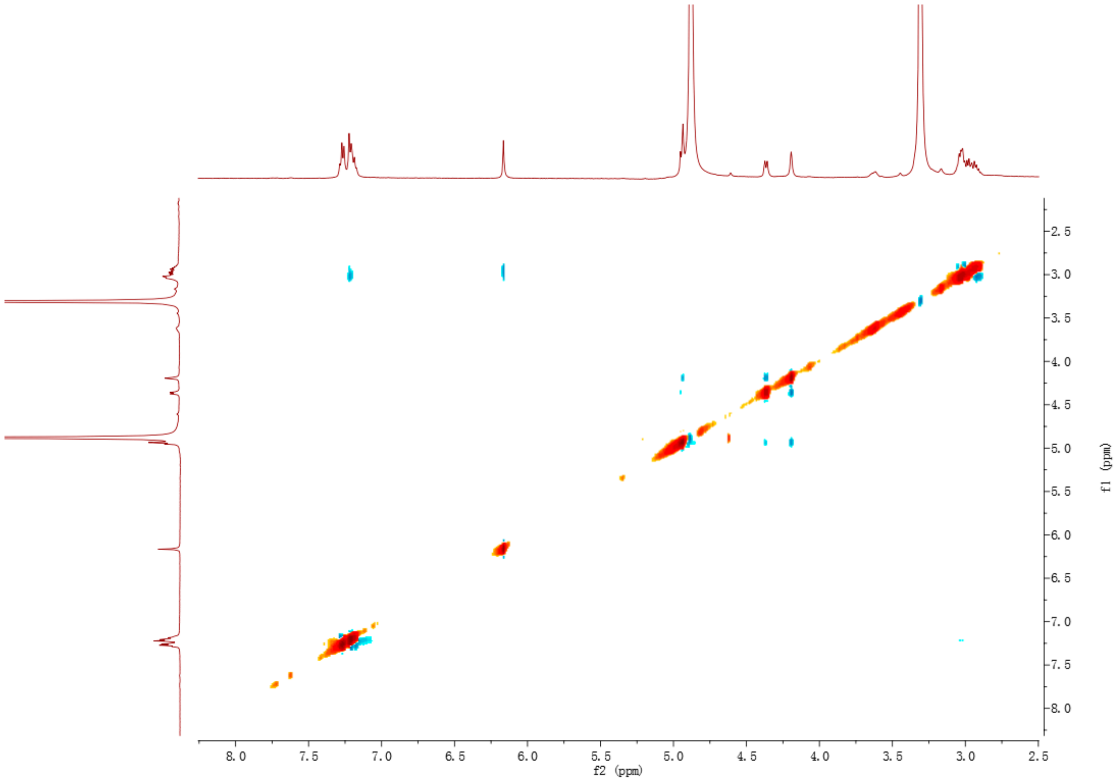

Figure S9. NOESY spectrum of Compound **1**.

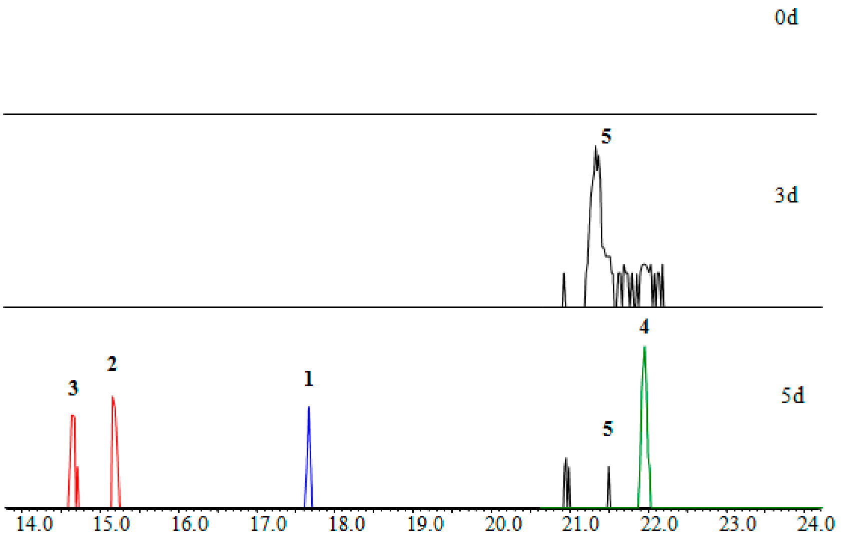

Figure S10. Extracted ion chromatogram of extracts from 0 d, 3 d, and 5 d NaCl-treated *A. sinensis* cell suspensions. The extracted ion chromatogram corresponding to Compound **1:** *m*/*z* 377.0318 $[M + Na]^+$, **2:** *m*/*z* 359.0657 $[M + Na]^+$, **3:** *m*/*z* 359.0657 $[M + Na]^+$, **4:** *m*/*z* 311.1278 $[M + H]^+$, and **5:** *m*/*z* 341.1384 $[M + H]^+$. Column: Agilent-ZORBAX SB-C_{18} (5 μm, 250 mm × 4.6 mm i.d.). Mobile phase: 0–30 min, 5–95% acetonitrile.

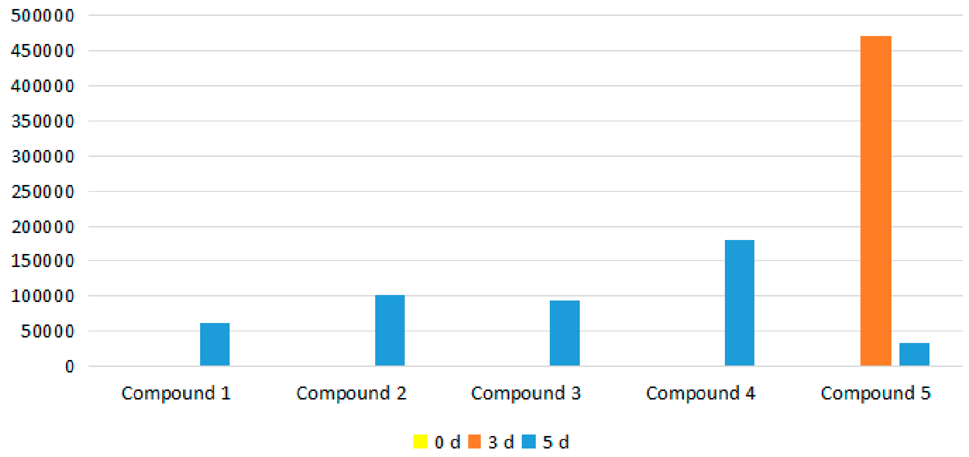

Figure S11. Accumulation of Compounds **1-5** in *Aquilaria sinensis* cell cultures treated with 150 mM NaCl for 0, 3 and 5 days. Relative production was calculated from the peak area presented in the extracted ion chromatograms.
